# Supplementary material for: Atomic structure and domain wall pinning in samarium-cobalt-based permanent magnets
Source: Nat Commun. 2017 Jul 4;8:54. doi: 10.1038/s41467-017-00059-9 (PMC5496909; doi:10.1038/s41467-017-00059-9)
Supplement: Supplementary file 1 — Supplementary Information [file 41467_2017_59_MOESM1_ESM.pdf]

File Name: Supplementary Information
Description: Supplementary Figures, Supplementary Table, Supplementary Note and 17 Supplementary References

File Name: Supplementary Movie 1
Description: Dynamical magnetization reversal process in Sm-Co with 19 wt.% Fe.

File Name: Supplementary Movie 2
Description: Dynamical magnetization reversal process in Sm-Co with 23 wt.% Fe.

File Name: Peer Review File
Description:

Supplementary information

1. Supplementary Note 1: Electron Microscopy

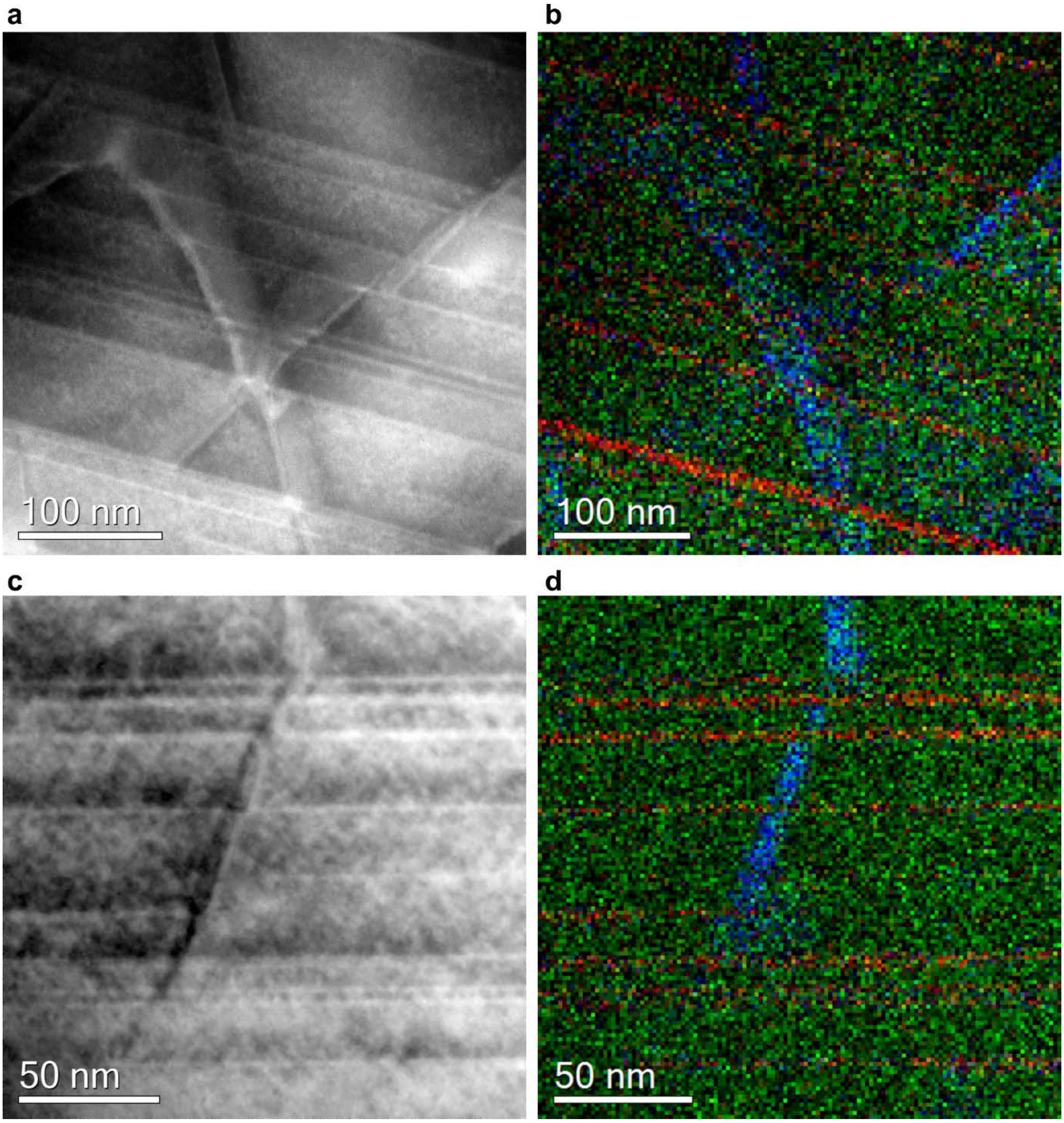

Supplementary Figure 1: STEM-HAADF images and EDX elemental maps. (a) STEM-HAADF image and (b) EDX elemental map (red=Zr-K, green=Sm-L, and blue=Cu-K) from sample 1. (c) STEM-HAADF image of sample 2 and (d) EDX elemental map (red=Zr-K, green=Sm-L, and blue=Cu-K). The single phases in the EDX maps can then be identified: (red) Z-phase, (green) 2:17 matrix, and (blue) 1:5 boundary phase.

Sample	Phase	Fe [at%]	Co [at%]	Cu [at%]	Zr [at%]	Sm [at%]
	k-factor	1.191	1.261	1.393	4.655	2.099
1	2:17 matrix	24.37 ± 1.68	55.98 ± 1.59	4.94 ± 2.19	1.47 ± 0.73	13.24 ± 0.85
	1:5 boundary phase	20.20 ± 4.56	52.52 ± 7.01	11.22 ± 10.52	2.73 ± 1.35	13.33 ± 1.80
	Z-phase	22.04 ± 1.85	55.78 ± 0.76	5.16 ± 1.17	3.96 ± 1.85	13.06 ± 0.88
2	2:17 matrix	27.69 ± 1.02	53.62 ± 0.84	3.84 ± 0.85	2.34 ± 0.86	12.51 ± 0.66
	1:5 boundary phase	25.67 ± 1.91	51.25 ± 2.67	8.32 ± 3.54	1.96 ± 0.33	12.79 ± 0.80
	Z-phase	27.40 ± 1.38	53.00 ± 1.37	4.88 ± 2.12	2.45 ± 1.28	12.29 ± 0.51

Supplementary Table 1: Quantitative TEM-EDX analysis of the single phases. The quantification was carried out standardless according to the Cliff-Lorimer method.

The microstructure of fully heat treated $Sm_2(Co, Fe, Cu, Zr)_{17}$ PM consists of three phases [1,2]: (i) The rhombohedral (space group R-3m) $Sm_2(Co, Fe)_{17}$ (2:17 matrix), (ii) a 5-10 nm thick Cu-rich, hexagonal (P6/mmm) cell boundary phase $Sm(Co,Cu)_5$ (1:5 boundary phase) dividing the 2:17 into 100-200 nm sized diamond-shaped cellular structure and (iii) a Zr-rich platelet phase (Z-phase) precipitating perpendicular to the hexagonal c-axis in high-coercivity magnets. [3–6]

The formation of a cell structure is essential for obtaining a high coercivity permanent magnet. The diamond-shaped cells, i.e. the 2:17 matrix, should be rich in Fe and Co, but depleted in Cu and Zr. The 1:5 cell boundary should be depleted in Fe and Co, but enriched in Cu and Sm. The lamellas are strongly enriched in Zr and depleted in Cu. Thus, the chemical composition of the phases has been investigated by EDX in the TEM. The results are shown in Supplementary Figure 1 and Supplementary Table 1. Supplementary Figure 1 (a) shows an ADF image of sample 1 where all important features are present, i.e. 2:17 matrix phase, 1:5 boundary phase, and the Z-phase. Supplementary Figure 1 (b) is an EDX elemental mapping of the same region. The Z-phase can be easily identified via the red color stemming from the Zr signal. The same holds for the 1:5 boundary phase displayed in blue, where Cu-K was used for fingerprinting. The 2:17 matrix is shown in green using the Sm-L signal. It is obvious that

Sm is distributed homogeneously in the 2:17 matrix phase as indicated by the green color in both images. Zr was found as expected to be enriched in the platelets as can be seen by the red color in both EDX maps. The Cu distribution showed a similar trend to enrich itself in the 1:5 cell boundary as indicated in the EDX maps by the blue color. Some regions close to the boundary phase are enriched in Cu, but depleted in Fe indicating that the diffusion process was not finished (fast cooling).

Supplementary Figure 3 shows the different types of twin interfaces being observed as well as the benefit of atomic resolution Z-contrast images compared to high-resolution phase contrast images. Supplementary Figure 3 (a) shows a high-resolution phase contrast image of a twin interface where just two orientations of a single phase are present having a fixed orientation relation to each other. Supplementary Figure 3 (b) shows a high-resolution phase contrast image of a twin interface with a Z-phase platelet lying in between the two twins. The inset images are image simulations. However, image interpretation with respect to atomic positions especially at the interface is difficult. Supplementary Figure 3 (c) is a STEM-HAADF Z-contrast image of a triple phase junction, i.e. of the 2:17 matrix, a Z-phase platelet, and a 1:5 boundary phase, with one twin on the lower left of the image and a second one on the upper right. Supplementary Figure 3 (d) shows the same type of area as in Supplementary Figure 3 (b), but acquired in C_s-corrected Z-contrast (STEM-HAADF) imaging mode the interpretation is straightforward since there is no contrast transfer reversal.

Another important point is that the coherency between the lattices of the Z-phase and the 2:17 matrix is maintained by minimization of the elastic misfit strain energy. We can confirm this assumption of Maury *et al.* [1] by a multiscale analysis approach in the TEM. No misfit dislocation strain fields at the Z-phase to 2:17 matrix interface were observed over several 100 nm in conventional bright-field images. Even on the atomic scale the lattice fit between both phases is remarkable as can be seen in Supplementary Figure 1. The lattice misfit between the Z-phase and the 2:17 matrix, which is approximately according to Maury *et al.* [1] about -1.3% in the basal plane and 2.6% along the *c*-axis is elastically compensated without forming any interfacial dislocations. Moreover, in the contribution of Maury *et al.* [1] the authors claim that it is more favorable to form new platelets rather than to grow existing ones along the *c*-axis of the 2:17 matrix. This is only partially true. In both samples we observed a distribution of Z-phase thicknesses, i.e. single stacks and multiple stacks. However, the Fe content has an influence on this behavior, since in the high Fe content sample incomplete formed Z-phase platelets were observed often in close proximity to

existing platelets. The structural integration of the 1:5 phase boundary into 2:17 matrix was already described. [7,8] They quote a lattice mismatch of 2.9% in *a*-direction and -2.5% in *c*-direction for the pure material. If Cu is introduced the lattice mismatch in *a*-direction increases, whereas it decreases in *c*-direction. Also in case of the 1:5 cell boundary phases we did not observe any lattice mismatch related dislocations in *c*-direction indicating a fully elastically strained material. Livingston and Martin [7] stated that because the lattice mismatches the *a*- and *c*-directions have opposite sign yielding a cone-like structure of zero misfit along which the 1:5 boundaries grow.

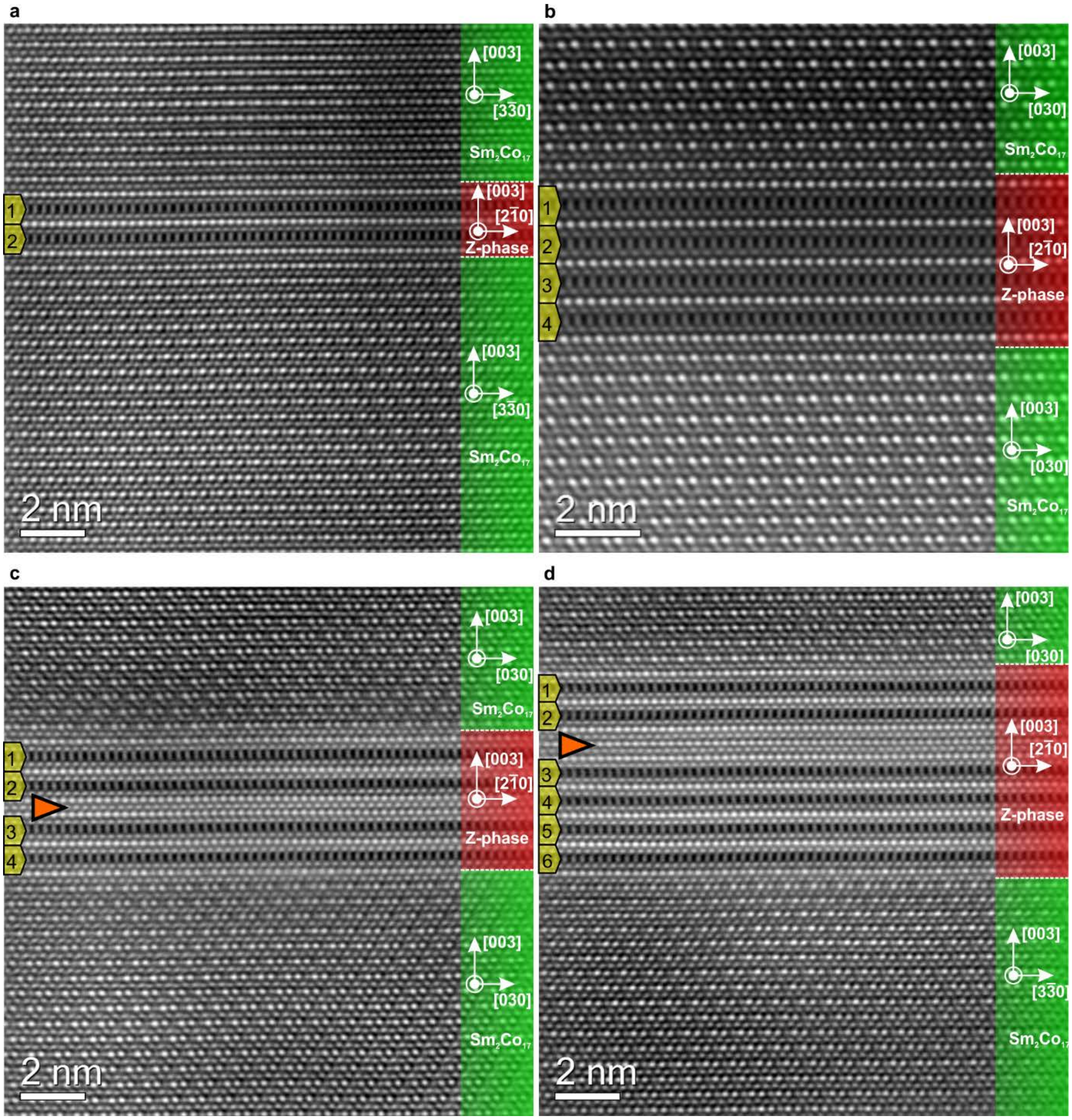

Supplementary Figure 2: Atomic resolution HAADF-STEM images of different Z-phase stacks oriented along the [120] zone axis. The number of stacks is denoted by the numbered yellow arrows. Dual (a) and quadruple (b) Z-phase stack. Faulted quadruple (c) and sextuple (d). Z-phase stacks with stacking faults are denoted by the orange triangle.

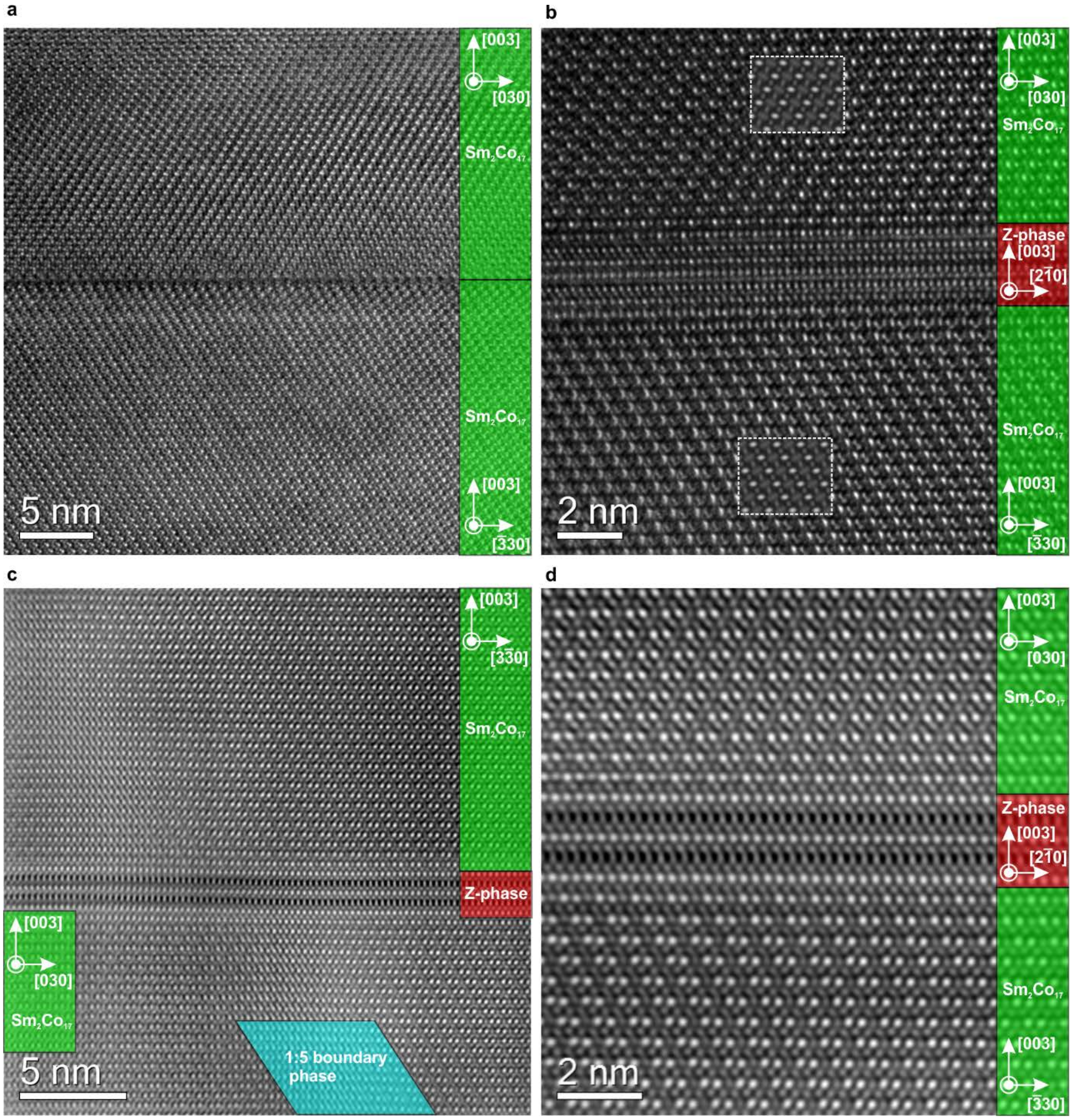

Supplementary Figure 3: High-resolution phase contrast TEM images and atomic resolution HAADF-STEM images. (a) A direct twin interface, i.e. the two 2:17 twins are in direct contact and (b) with a Z-phase platelet in between. The insets in (c) show image simulations of the 2:17 matrix for the two different orientations. Atomic resolution Z-contrast images of (d) a twin interface with a 1:5 boundary interface in between and (d) with a Z-phase platelet. All structural features shown here are from sample 1, but are also valid for sample 2 except for the 1:5 type cell boundaries.

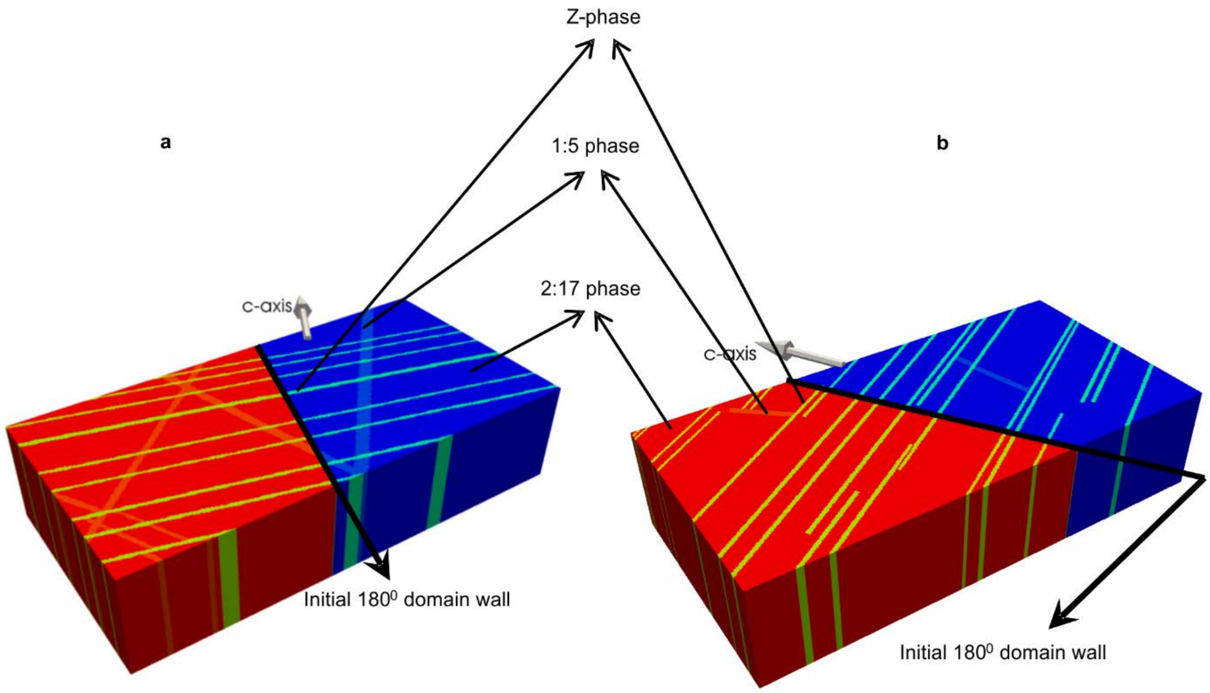

Supplementary Figure 4: Micromagnetic models. (a) Sample 1 and (b) sample 2. These models are with a size of 440×440×220 nm^3, which only include the upper half of the microstructure in the TEM images (Figure 1 in the main text). The 1:5 phase, 2:17 phase, and Z-phase are indicated by the arrows. An initial 180 degree domain wall is set along the easy axis. The external magnetic field antiparallel to the c-axis is applied to stimulate the domain wall movement.

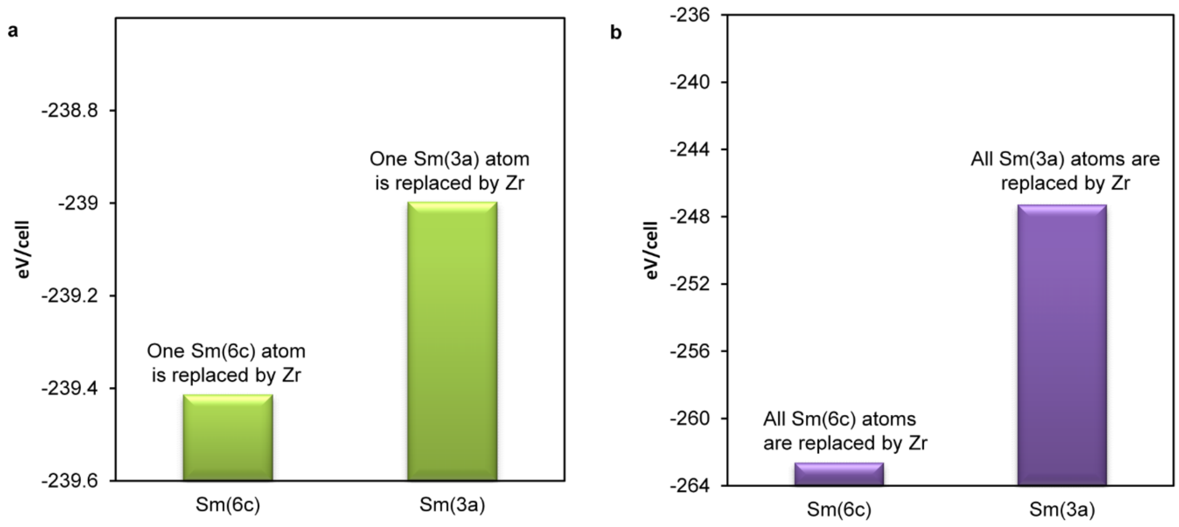

Supplementary Figure 5: Site-preference energies for Zr doping. (a) Energy per unit cell when one Sm 1(6c) or Sm 2(3a) atom is replaced by one Zr atom in $SmCo_3$. (b) Energy per unit cell when all the Sm 1(6c) or Sm 2(3a) atoms are replaced by Zr atoms in $SmCo_3$. Comparison of energy in (a) and (b) shows that Sm1 (6c) is energetically favorable.

Supplementary References

1. Maury, C., Rabenberg, L. & Allibert, C. H. Genesis of the cell microstructure in the Sm(Co, Fe, Cu, Zr) permanent magnets with 2:17 type. *Phys. Status Solidi A* **140,** 57–72 (1993).

2. Xiong, X. Y. *et al.* The microstructure of sintered $Sm(Co_{0.72}Fe_{0.20}Cu_{0.055}Zr_{0.025})_{7.5}$ permanent magnet studied by atom probe. *Acta Mater.* **52,** 737–748 (2004).

3. Rabenberg, L., Mishra, R. K. & Thomas, G. Microstructures of precipitation-hardened SmCo permanent magnets. *J. Appl. Phys.* **53,** 2389–2391 (1982).

4. Fidler, J., Skalicky, P. & Rothwarf, F. High resolution electron microscope study of $Sm(Co, Fe, Cu, Zr)_{7.5}$ magnets. *IEEE Trans. Magn.* **19,** 2041–2043 (1983).

5. Schrefl, T., Fidler, J. & Scholz, W. Modeling and limits of advanced HT-magnets. *IEEE Trans. Magn.* **36,** 3394–3398 (2000).

6. Yang, J., Shindo, D. & Hiroyoshi, H. Observation of Microstructures and Magnetic Domains of Sm-Co Based Permanent Magnets by High-Voltage Electron Microscopy. *Mater. Trans. JIM* **38,** 363
7. Livingston, J. D. & Martin, D. L. Microstructure of aged $(Co,Cu,Fe)_7Sm$ magnets. *J. Appl. Phys.* **48,** 1350–1354 (1977).
8. Melton, K. N. & Perkins, R. S. Magnetic properties of Sm: (Co,Cu) alloys. I. Electron microscopy. *J. Appl. Phys.* **47,** 2671–2678 (1976).
